# Differential Impact of Co-expressed SP-A1/SP-A2 Protein on AM miRNome; Sex Differences

**DOI:** 10.3389/fimmu.2019.01960

**Published:** 2019-08-16

**Authors:** Nithyananda Thorenoor, Yuka Imamura Kawasawa, Chintan K. Gandhi, Xuesheng Zhang, Joanna Floros

**Affiliations:** ^1^Department of Pediatrics, Center for Host Defense, Inflammation, and Lung Disease Research, The Pennsylvania State University College of Medicine, Hershey, PA, United States; ^2^Departments of Pharmacology and Biochemistry and Molecular Biology, Institute for Personalized Medicine, The Pennsylvania State University College of Medicine, Hershey, PA, United States; ^3^Department of Obstetrics and Gynecology, The Pennsylvania State University College of Medicine, Hershey, PA, United States

**Keywords:** surfactant protein A, alveolar macrophages, miRNA, surfactant protein A1/A2, sex differences, oxidative stress

## Abstract

In humans there are two surfactant protein A (SP-A) functional genes *SFTPA1* and *SFTPA2* encoding innate immune molecules, SP-A1 and SP-A2, respectively, with numerous genetic variants each. SP-A interacts and regulates many of the functions of alveolar macrophages (AM). It is shown that SP-A variants differ in their ability to regulate the AM miRNome in response to oxidative stress (OxS). Because humans have both SP-A gene products, we were interested to determine the combined effect of co-expressed SP-A1/SP-A2 (co-ex) in response to ozone (O_3_) induced OxS on AM miRNome. Human transgenic (hTG) mice, carrying both SP-A1/SP-A2 (6A^2^/1A^0^, co-ex) and SP-A- KO were utilized. The hTG and KO mice were exposed to filtered air (FA) or O_3_ and miRNA levels were measured after AM isolation with or without normalization to KO. We found: (i) The AM miRNome of co-ex males and females in response to OxS to be largely downregulated after normalization to KO, but after Bonferroni multiple comparison analysis only in females the AM miRNome remained significantly different compared to control (FA); (ii) The targets of the significantly changed miRNAs were downregulated in females and upregulated in males; (iii) Several of the validated mRNA targets were involved in pro-inflammatory response, anti-apoptosis, cell cycle, cellular growth and proliferation; (iv) The AM of SP-A2 male, shown, previously to have major effect on the male AM miRNome in response to OxS, shared similarities with the co-ex, namely in pathways involved in the pro-inflammatory response and anti-apoptosis but also exhibited differences with the cell-cycle, growth, and proliferation pathway being involved in co-ex and ROS homeostasis in SP-A2 male. We speculate that the presence of both gene products vs. single gene products differentially impact the AM responses in males and females in response to OxS.

## Introduction

Surfactant protein A (SP-A) plays important role in lung innate immunity and surfactant-related functions under basal conditions (1–5) and in response to various insults such as infection and oxidative stress (6–10). The human SP-A locus consists of two functional genes, *SFTPA1* and *SFTPA2*, and one pseudogene (11, 12). The functional genes encode human SP-A1 and SP-A2 proteins, respectively, and each gene has been shown to have several genetic and splice variants (13, 14).

Human SP-A is expressed in alveolar epithelial type II cells (15) and in other tissues (16–19). It has been reported that human SP-A exists as octadecamer with six trimers (20), and that SP-A trimers have two SP-A1 molecules, and one SP-A2 molecule in the ratio of 2:1 (21). Previous studies from our group and others have shown that the ratio of the proposed model at mRNA and protein levels varies (22, 23). In bronchoalveolar lavage (BAL) fluid the ratio between SP-A1 to total SP-A varies as a function of age and health status (23, 24). The SP-A1 and SP-A2 mRNA content was found to vary in explant cultures under different conditions (25–27). Moreover, more SP-A2 mRNA than SP-A1 was observed in lung tissues of adults, whereas more SP-A1 mRNA transcripts were detected in neonates (28).

Several studies demonstrated that single gene products, SP-A1 and SP-A2, exhibit both qualitative (i.e., functional, biochemical and/or structural) (29–43), and quantitative (regulatory) differences (23, 25–27, 44–46). For example, SP-A1 and SP-A2 variants have been shown to differ in their ability to modulate the proteomic expression profile of AM and the AM actin cytoskeleton (47–49). The proteome profile of AM from KO mice, after treatment with exogenous SP-A1 or SP-A2 resulted in significant changes in proteins involved in the oxidative stress response pathway, with females being more responsive to SP-A1 and males to SP-A2 (48), as well as the single cell analysis revealed sex- and age-related differences in alveolar macrophage phenotypes from KO mice in response to SP-A1 and SP-A2 proteins (49). Moreover, sex differences have been observed between SP-A1 and SP-A2 and among variants in survival and lung function mechanics in response to bacterial infection (42, 43), and SP-A1 compared to SP-A2 exhibits a higher efficiency in pulmonary surfactant reorganization (50). The major contributor for at least some of these differences appears to be amino acid 85 of the precursor molecule; where SP-A1 has a cysteine and SP-A2 has an arginine (14). This Cys/Arg is a key difference between SP-A1 and SP-A2. This single amino acid change has a major impact on SP-A oligomerization, lipopolysaccharide (LPS) aggregation, and phagocytosis (38). The replacement of cysteine of SP-A1 with arginine or the arginine of SP-A2 with cysteine resulted in a reversal pattern of SP-A oligomerization and functional activity of both mutants of SP-A1 and SP-A2 (38). Thus, structural differences due to Cys85 and other amino acids may underlie differences in function observed between SP-A1 and SP-A2.

Ozone (O_3_), a major component of air pollution and a strong oxidizing agent known to cause toxicity in the lower airways, has significant effects on innate host defense and lung function (51). The O_3_ exposure can cause, edema, contributing to lung injury, and pulmonary surfactant derangement (52). A significant difference in survival has been observed with females being more affected than males in several lung diseases (53–56). In our animal studies, we observed significant differences in survival after infection and O_3_ exposure, with females being more susceptible to oxidative stress than males (7, 9) and sex hormones have been implicated in the observed differences in survival (57) but the mechanism underlying these differences is unknown. Moreover, during pneumonie infection and bacterial clearance, the ability to limit bacterial dissemination, and the phagocytic activity of alveolar macrophages may play an important role in the differential outcome in survival between males and females in the presence or absence of oxidative stress (7, 9, 58). Previously, we observed significant changes in AM miRNome of SP-A2 males but not in SP-A2 females or in SP-A1 males and females in response to OxS (41).

In the present study, we investigated the hypothesis that male and female mice expressing both SP-A1/SP-A2 gene products (co-ex) differentially regulate the AM miRNome in response to ozone-induced oxidative stress and that this differs from that previously observed in SP-A single gene variants (41). Toward this co-ex male and female mice were exposed to filtered air (FA) or O_3_ and the expression levels of 307 miRNAs was measured with or without normalizing to miRNAs identified from KO under the same conditions. We found significant differences in the AM miRNome of co-ex in terms of sex, exposure, with or without normalization to KO. Comparison of the co-ex miRNome to that of hTG mice carrying SP-A2 variant showed that the pathways involved in AM SP-A2 share some similarities to that of co-ex, but also exhibit differences.

## Methods

### Animals

Humanized transgenic (hTG) mice carrying both gene variants, SP-A1/SP-A2 (6A^2^/1A^0^, co-ex), as well as SP-A knockout (KO) mice were used in this study. They were 12 weeks old. hTG mice were generated on the C57BL/6J SP-A (KO) background (59). The animals used in this study were raised and maintained in a pathogen-free environment, at the Penn State College of Medicine animal facility as described previously (43). Both males and females were used. The females were synchronized with regard to the estrous cycle as described previously (43). A total of 44 mice (32 for miRNA analysis and 12 for qRT-PCR analysis) were used in the present study. All the procedures were approved by The Penn State Hershey Medical Center Institutional Animal Care and Use Committee (IACUC).

### Filtered Air (FA) and Ozone (O_3_) Exposure

The animals were exposed to FA or O_3_ in parallel as described previously (60). A group of 4 animals (males, females) were exposed to FA or O_3_ for 3 h, and alveolar macrophages (AM) were isolated after 4 h of recovery as described (61).

### RNA Preparation, Library Construction, and Sequencing

Total RNA was extracted from AMs using mirVana kit (#AM1560, Ambion, Waltham, MA). The extracted RNAs were quantified and quality checked using a BioAnalyzer RNA 6000 Nano Kit (Agilent Technologies, Santa Clara, CA). Small RNA-seq libraries were generated by NEXTflex Small RNA Library Prep Kit v3 for Illumina (BioO Scientific, Austin, TX), followed by deep sequencing on an Illumina HiSeq 2500 as per the manufacturer's instructions. Briefly, 1-2 ng of total RNA was ligated with chemically modified 3′- and 5′- adapters that can specifically bind to mature micro RNAs, followed by reverse transcription and PCR amplification. Unique index sequence tags were introduced during PCR to enable multiplexed sequencing. Each library was assessed for the presence of desired micro RNA population and approximate library quantity by Bioanalyzer High Sensitivity DNA Kit (Agilent Technologies). Pooled libraries were denatured and loaded onto a TruSeq Rapid flow cell on an Illumina HiSeq 2500 and run for 50 cycles using a single-read recipe according to the manufacturer's instructions. De-multiplexed sequencing reads passed the default purify filtering of Illumina CASAVA pipeline (released version 1.8) and were quality trimmed/filtered using The FASTX-Toolkit (http://hannonlab.cshl.edu/fastx_toolkit). The filtered reads were further trimmed with both 5′ and 3′ adapter sequences and subjected to Chimira suite to align and count miRNA expression (62). The differentially expressed miRNAs (DEG) between FA to O_3_, males and females were identified by using the edgeR (63) and the TCC v1.14.0 R package (64) with false discovery rate (FDR) adjusted *p*-value of 0.1 as a significance cutoff.

### miRNA Data Analysis

We successfully identified 307 miRNAs with good correlation between mice (3 out of 4, Supplementary Table 1). We used two different parameters (methods) to analyze the identified miRNA expression (fold change). (a) The expression levels (fold change) of miRNAs from co-ex (FA to O_3_ for males and females) and KO (FA to O_3_ for males and females) were analyzed and differentially expressed miRNAs in co-ex and KO males and females were identified in response to FA or O_3_. (b) The changes in miRNA expression in co-ex were calculated by normalizing to KO, i.e., the level of expression of each individual experimental miRNA in co-ex males and females exposed to FA or O_3_ was divided by the corresponding miRNA in the KO. Next, the differentially expressed miRNAs between co-ex males and females were determined by dividing a specific individual male miRNA by the corresponding female miRNA (Supplementary Table 1).

### Gene Expression Analysis

The expression levels of CCND1, CCND2, CCNE1, CDK7, IL-6, IL-10, TLR2, TLR3, STAT3, MYD88, IL-4, IL2RG, EGR2, PTEN, TNFSF12, MDTH, JUN, E2F3, BCL2, TNF, CDK2, SMAD2, MMP2, ARG1, AKT1, PPARA, and MYC genes at mRNA level in the female and male co-ex and KO AM, were validated by qRT-PCR as described previously (41). The RT2 qPCR Primer assays were purchased from Qiagen. The AM cell samples [3 animals/treatment (FA or O_3_)] were analyzed in triplicates/animal and quantified relative to GAPDH mRNA.

### Statistical Analysis

Statistical differences between miRNA expression levels (fold change) in FA vs. O_3_ and male vs. female were evaluated by two-tailed *t*-test and nonparametric Mann-Whitney test. For multiple comparison analysis one-way analysis of variance (ANOVA) was employed followed by Bonferroni multiple comparisons. The *p*-values <0.05 were considered to be significant. All the data points are means ± standard deviation, and analyses were performed using Graph-Pad Prism software version 5.0 (Graph-Pad Software, San Diego, USA).

## Results

We have previously studied the AM miRNome in hTG mice carrying either SP-A1 or SP-A2 variants using the qRT-PCR method (41). In this study, because humans have both SP-A gene products, we were interested to a) determine the combined effect of SP-A1/SP-A2 (6A^2^/1A^0^, co-ex) on the AM miRNome in response to oxidative stress with or without normalization to KO in males and females; b) identify sex, treatment, and gene (co-ex) impact on the AM miRNome; c) use differentially expressed miRNAs of ≥2 fold in co-ex in response to O_3_ exposure in Ingenuity Pathway Analysis (IPA) to identify biological functions and regulatory network targets of the identified and differentially expressed miRNAs after normalized to KO and Bonferroni multiple corrections; d) compare the co-ex miRNome (present study) and its targets to that of hTG mice carrying SP-A2 (41) variant under the same condition.

### Effect of SP-A1/SP-A2 (6A^2^/1A^0^, co-ex) on AM miRNome Regulation

#### Without Normalization to KO

**Figure 1:**
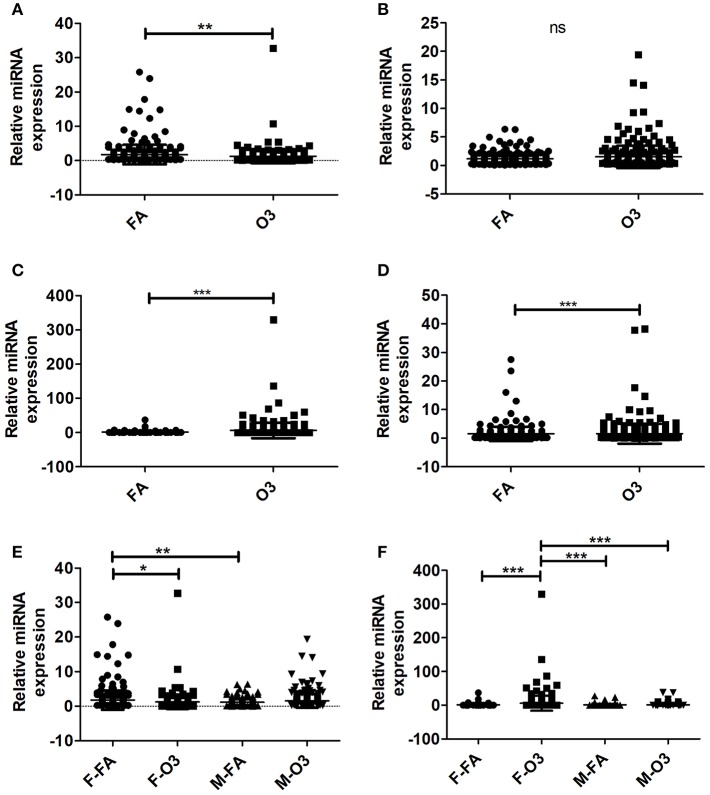
Regulation of the AM miRNome in co-ex and KO males (M) and females (F) after Filter air (FA) and Ozone (O_3_) exposure. Comparisons between miRNAs identified after FA or O_3_ exposure in co-ex females **(A)**, males **(B)**, and KO females **(C)**, males **(D)**. Significant differences observed between treatment in co-ex females, KO males and females (**A,C,D**; *P* < 0.05). **(E,F)** Depict the Bonferroni multiple comparisons of the miRNAs identified after FA or O_3_ exposure in co-ex and KO, respectively. A significant difference is observed in co-ex and KO females as a function of treatment **(E,F)**. Significant differences (*P* < 0.05) in miRNA regulation were observed between sexes in co-ex (F-FA vs. M-FA, **E**), and in KO (F-O_3_ vs. M-O_3_, F-O_3_ vs. M-FA, **F**). ns, not significant. ^*^p < 0.05, ^**^p < 0.001, ^**^p < 0.0001.

A total of 307 miRNAs from AMs of co-ex and KO in response to FA or O_3_ were identified from males and females (Supplementary Table 1). We observed significant differences (*p* < 0.05) in the expression of AM miRNAs in response to FA or O_3_ in co-ex females and KO males and females (Figures 1A,C,D). No significant differences were observed in co-ex males, after exposure to FA or O_3_ (Figure 1B). One-way ANOVA and Bonferroni multiple comparison analysis was performed to find the effect of treatment (FA, and O_3_) and sex (males, and females) as well as the interaction between the two parameters (sex and treatment). In co-ex, there was a significant difference between FA-exposed males and females (Figure 1E) but no significant difference was observed after O_3_ exposure between males and females (Figure 1E). However, females (but not males) showed a significant difference between FA or O_3_ exposure (Figure 1E). Whereas, in KO (i.e., in the absence of SP-A altogether), there was a significant difference between male and female after O_3_ exposure, no significant difference was observed after FA exposure (Figure 1F), indicating a role of SP-A in O_3_ exposure between sexes. However, similar to what was shown for the co-ex (Figure 1E), the KO females (but not the males) showed a significant difference after FA or O_3_ exposures (Figure 1F). Moreover, the miRNAs identified from KO females exposed to O_3_, showed a significant difference compared to KO males exposed to FA (Figure 1F).

#### Normalization to KO

**Figure 2:**
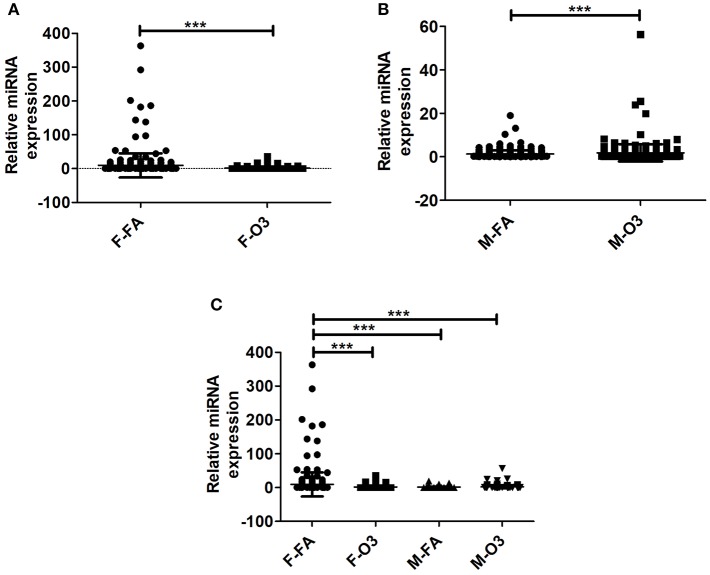
Effect of normalization on the regulation of miRNAs. The miRNAs identified from co-ex were normalized to same miRNAs identified in KO males and females after FA or O_3_ exposure. **(A,B)** Depict the differences in miRNA regulation in females and males after FA or O_3_. Significant differences (*P* < 0.05) were observed in both sexes after FA or O_3_ exposure **(A,B)**. **(C)** Depicts the Bonferroni multiple comparisons of the miRNAs identified after FA or O_3_ exposure in co-ex. A significant difference (*P* < 0.05) was observed between co-ex FA or O_3_ exposed females and between sexes in co-ex (F-FA vs. M-FA, and F-FA vs. M-O_3_) **(C)**. ^***^p < 0.0001.

By normalizing the expression of miRNAs in co-ex to KO, i.e., the level of expression of each individual experimental miRNA (i.e., in SP-A1/SP-A2, co-ex) was divided by the level of the corresponding miRNA in the KO (Supplementary Table 1), we found significant differences in the differential expression of miRNAs in both males and females after FA or O_3_ exposure (Figures 2A,B). The one-way ANOVA and Bonferroni multiple comparison analysis resulted in similar observations as those observed in Figure 1E in the absence of KO normalization. Significant differences in miRNAs differentially expressed were observed in females between FA to O_3_ exposure and FA-exposed females to FA-exposed males, with no significant difference observed of differentially expressed miRNAs after O_3_ exposure between males and females (Figure 2C). Unlike in Figure 1E, FA-exposed females differed significantly from O_3_-exposed males (Figure 2C).

The observations made with or without normalization to KO indicate that the AM miRNome of hTG mice carrying both SP-A1/SP-A2 (6A^2^/1A^0^, co-ex), exhibit no differences in miRNA expression between sexes in response to oxidative stress (O_3_ exposure), but sex differences are observed in controls (i.e., after FA exposure; Figures 1E, 2C). Female co-ex exhibited significant differences between FA or O_3_ exposure. The presence of the two genes may play a protective role in the outcome of miRNA expression in response to oxidative stress, especially in males.

### Regulation of miRNAs That Changed ≥2 Fold in Response to FA, O_3_, and Sex

We further looked into the AM miRNAs, whose expression compared to control miRNAs is altered ≥2 fold in response to FA or O_3_ from co-ex and KO males and females, and compared them between males and females.

#### Without Normalization to KO

**Figure 3:**
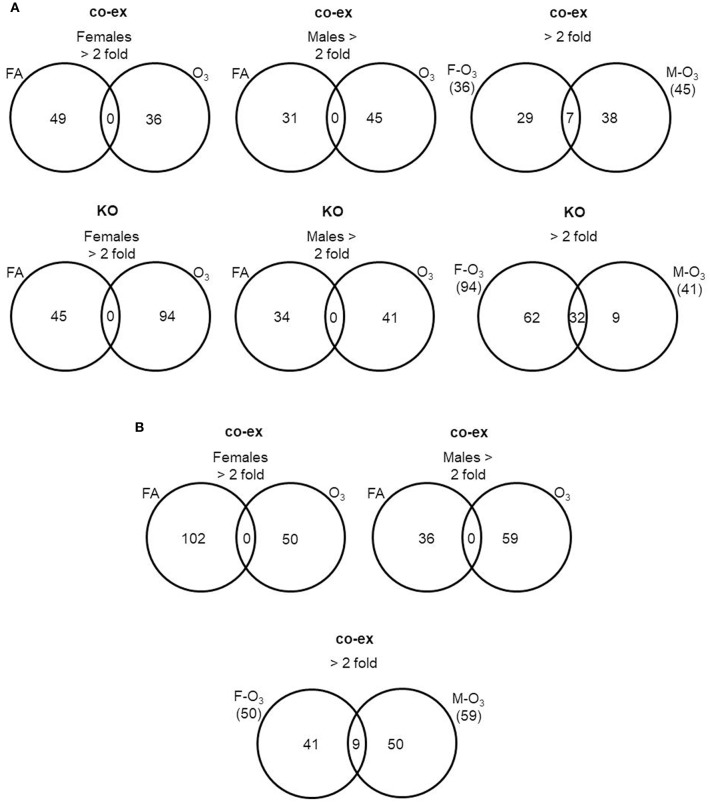
Comparison of miRNAs affected by FA or O_3_ in co-ex females and males. A. The Venn diagrams show miRNAs with significant changes ≥2 fold in response to FA or O_3_ in AM of males and female co-ex and KO. Out of the 307 miRNAs identified after FA or O_3_, 49 miRNAs had ≥2 fold and 36 miRNAs were ≥2 fold after FA or O_3_ exposure respectively, in co-ex females and in co-ex males 31 miRNAs had ≥2 fold and 45 miRNAs were ≥2 fold after FA or O_3_ exposure **(A)**. A similar comparison in KO resulted in 45 miRNAs with ≥2 fold and 94 miRNAs with ≥2 fold after FA or O_3_ exposure, respectively, in KO females, and in KO males 34 miRNAs and 41 miRNAs with ≥2 fold after FA or O_3_ exposure, respectively. In both co-ex and KO there were no differentially regulated miRNAs found in common after FA or O_3_ exposure **(A)**. A comparison of differentially regulated miRNAs after O_3_ exposure in co-ex between females and males, identified 36 miRNAs ≥2 fold in females and of these 29 were specific to females. In males 45 miRNAs ≥2 fold were identified and of these 38 were specific to males. Seven miRNAs were identified to be in common between females and males after O_3_ exposure **(A)**. Whereas, in KO of the 94 miRNAs ≥2 fold in females, 62 were specific to females, and of the 41 miRNAs ≥2 fold in males, 9 were specific to males. Thirty-two miRNAs were identified to be in common between females and males after O_3_ exposure **(A)**. **(B)** The Venn diagrams show the number of miRNAs identified from co-ex after normalization to the same miRNAs identified in KO males and females after FA or O_3_ exposure. Out of the 307 miRNAs identified after FA or O_3_, 102 miRNAs ≥2 fold and 50 miRNAs ≥2 fold were observed after FA or O_3_ exposure, respectively, in co-ex females. In co-ex males, 36 miRNAs ≥2 fold and 59 miRNAs ≥2 fold were identified after FA or O_3_ exposure, respectively. In both co-ex females and males, there were no differentially regulated miRNAs found in common after FA or O_3_ exposure. A comparison of differentially regulated miRNAs after O_3_ exposure in co-ex between females and males, identified 50 miRNAs ≥2 fold in females and of these, 41 were specific to females. In males of the 59 miRNAs ≥2 fold, 50 were specific to males and 9 miRNAs were identified to be in common between females and males after O_3_ exposure.

First we compared AM miRNAs of FA vs. O_3_ exposed animals. We found that in co-ex females, 49 miRNAs were changed after FA and 36 miRNAs after O_3_ exposure, whereas in males, 31 miRNAs were changed in FA and 45 in O_3_ exposure. The same comparison in KO AM miRNome showed that in females 45 miRNAs were changed ≥2 fold in FA and 94 miRNAs in O_3_, while in males 34 miRNAs were changed ≥2 fold in FA and 41 miRNAs in O_3_ (Figure 3A, Supplementary Table 1). In both co-ex and KO, all the miRNAs that had ≥2 fold are specific either to FA or O_3_ exposure, with no miRNAs in common between FA or O_3_ in either males or females.

Next, we compared males vs. females (co-ex, KO) in response to oxidative stress. In co-ex females, 36 miRNAs were differentially expressed (≥2 fold) compared to 45 miRNAs in males, with 7 miRNAs (≥2 fold) being in common in both males and females. Of these, 29 miRNAs are unique to females and 38 are unique to males (Figure 3A). In KO females after O_3_ exposure, 94 miRNAs had ≥2 fold expression compared to 41 miRNAs in males. Of these 32 miRNAs were found in common in both sexes (Figure 3A), and 62 miRNAs are unique to females and 9 to males (Figure 3A). Of the seven miRNAs found to be in common between co-ex male and females after O_3_ exposure only one miRNA is included in the 32 miRNAs found in common between KO males and females (Supplementary Table 1). Of interest the co-ex had a significantly lower number of shared miRNAs between the sexes compared to KO (7 vs. 32), indicating the major effect of sex on co-ex (Supplementary Table 1).

#### With Normalization to KO

By normalizing the miRNAs identified in co-ex to KO, we found 102 miRNAs to be differentially expressed ≥2 fold in females after FA exposure compared to 50 miRNAs after O_3_ exposure (Figure 3B). In the case of males, 36 miRNAs are differentially expressed after FA exposure and 59 miRNAs had ≥2 fold after O_3_ exposure. In both co-ex males and females, all the miRNAs with ≥2 fold are specific to either FA or O_3_. A comparison of miRNAs from males and females after O_3_ exposure showed 50 miRNAs ≥2 fold in females vs. 59 miRNAs in males (Figure 3B). Of these, 41 are unique to females and 50 to males, with 9 being found in common. These data indicate that when the expression of the AM miRNome in co-ex, after normalization to KO is compared in males and females, following exposure to FA or O_3_, the observed changes in miRNA levels are either specific to males or females. However, when the miRNome between males and females co-ex normalized KO is compared after oxidative stress there are few miRNAs that are found in common in co-ex males and females (Supplementary Table 1).

### Ingenuity Pathway Analysis (IPA)

**Figure 4:**
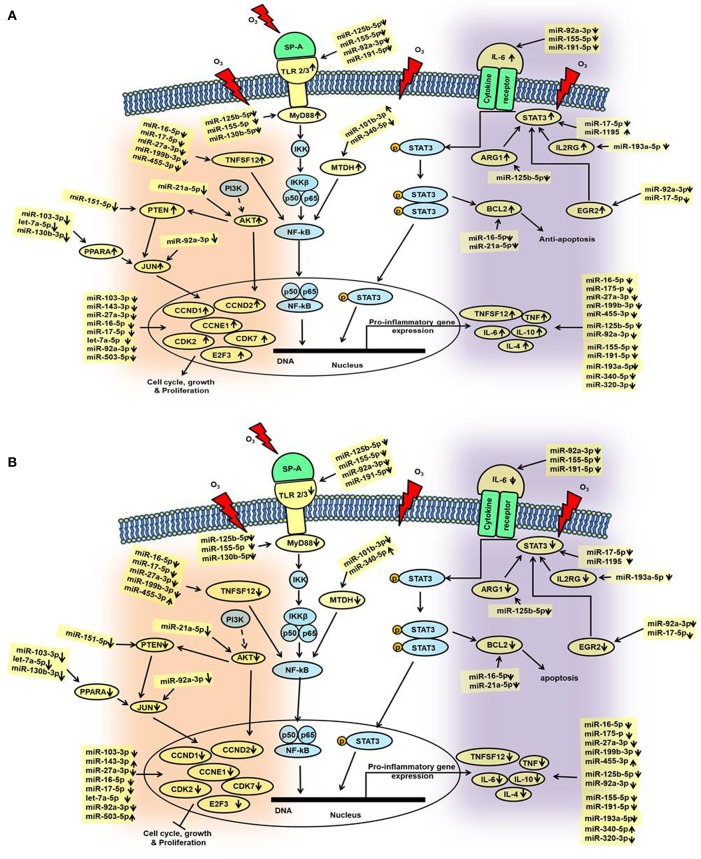
Schematic representation of the identified miRNAs in co-ex AM and their targets in response to OxS. This diagram depicts the significantly changed miRNAs and their targets in the various pathways. These include pathways of cell cycle, and cellular growth and proliferation as well as, pathways of pro-inflammatory response and anti-apoptosis, **(A)** males and **(B)** females. The miRNAs and genes studied in the present study are highlighted with yellow. Up (↑) and down (↓) arrows indicate increase or decrease.

We performed IPA to further understand the role of significantly changed miRNAs in co-ex males and females under the studied conditions. After Bonferroni correction, expression of miRNAs in co-ex females exhibited significant differences in response to OxS, but not in males (Figure 2C). However, the IPA analyses before and after the Bonferroni correction were identical because the miRNA data input in IPA was same. We were able to identify biological functions and regulatory network targets of these differentially expressed miRNAs. The targets shown in Figure 4 are involved in anti-apoptosis, cell cycle, cellular growth and proliferation, as well as pro-inflammatory response are affected by differential expression of miRNAs in response to O_3_ exposure (Supplementary Table 1). These targets include CCND1, CCND2, CCNE1, CDK7, IL-6, IL-10, TLR2, TLR3, STAT3, TNFSF12, MYD88, IL-4, IL2RG, EGR2, PTEN, MDTH, JUN, E2F3, BCL2, TNF, CDK2, MYC, SMAD2, MMP2, ARG1, AKT1, and PPARA mRNAs. The miRNAs that were changed significantly in co-ex females and males in response to O_3_ exposure and their targets are listed in Table 1.

**Table 1:** Expression levels of co-ex AM miRNAs (males and females) in response to OxS and its mRNA targets identified by IPA analysis.

**miRNA ID**	**Fold change in females**	**Fold change in males**	**Target molecule**
let-7a-5p	1.089^*^	1.327^*^	CCND1, CCND2, E2F3, PPARA
miR-101b-3p	1.152^*^	2.338	MTDH
miR-103-3p	0.819^*^	0.845^*^	E2F3, PPARA
miR-125b-5p	1.269^*^	0.834^*^	TLR2, TNF, ARG1, MYD88
miR-143-3p	2.370	0.835^*^	E2F3
miR-151-5p	1.127^*^	1.452^*^	PTEN
miR-155-5p	1.248^*^	1.316^*^	IL-6, TLR2, MYD88
miR-16-5p	1.148^*^	1.012^*^	CCND1, CCNE1, CDK7, TNFSF12, E2F3, BCL2
miR-17-5p	0.959^*^	0.952^*^	CCND1, CCND2, CCNE1, CDK7, STAT3, EGR2, E2F3, MYC, TNFSF12
miR-181a-5p	0.577^*^	1.053^*^	SMAD2
miR-191-5p	1.032^*^	0.455^*^	IL-6, TLR3
miR-193a-5p	0.252^*^	0.856^*^	IL-10, IL2RG
miR-199b-3p	1.829^*^	0.542^*^	PTEN, TNFSF12
miR-21a-5p	1.739^*^	1.447^*^	BCL2, AKT
miR-27a-3p	1.053^*^	1.482^*^	E2F3
miR-340-5p	2.572	1.051^*^	MTDH, MYD88
miR-455-3p	2.776	0.491^*^	TNFSF12
miR-503-5p	2.332	0.918^*^	CDK2
miR-92a-3p	0.773^*^	0.795^*^	CCND1, CCNE1, CDK7, IL-6, TLR2, TLR3, EGR2, JUN, E2F3, TNF, SMAD2
miR-1195	0.797^*^	4.739	STAT3
miR-320-3p	0.700^*^	0.876^*^	MYD88
miR-130b-3p	0.127^*^	0.505^*^	PPARA
miR-130b-5p	0.581^*^	0.445^*^	MYD88

* *Indicates downregulation*.

In general, we observed largely a downregulation of miRNAs in both males and females and upregulation of their targets in co-ex males but not in females (Figures 4A,B). For example, a significant downregulation of miR-191-5p, miR-155-5p, and miR-92a-3p expression was observed in response to O_3_ in both co-ex males and females but their target IL-6 mRNA is upregulated in males but downregulated in females. Though most of the miRNAs are downregulated in both males and females, the expression of miR-340-5p, miR455-3p, miR-143-3p, and miR-503-5p in females, and miR1195 and miR101b-3p in males is upregulated (Figures 4A,B).

### Validation of miRNA Target Genes

**Figure 5:**
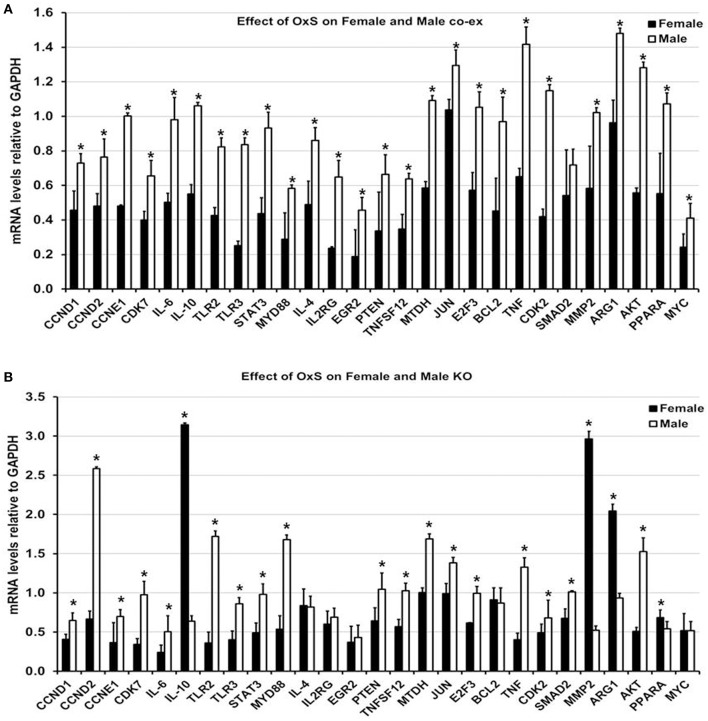
Effect of OxS on mRNA targets of co-ex and KO. **(A)** The expression of level of CCND1, CCND2, CCNE1, CDK7, IL-6, IL-10, TLR2, TLR3, STAT3, MYD88, IL-4, IL2RG, EGR2, PTEN, TNFSF12, MTDH, JUN, E2F3, BCL2, TNF, CDK2, MMP2, ARG1, AKT1, PPARA, and MYC, were significantly upregulated in co-ex males compared to females. The level of SMAD2 did not change between males and females. The expression levels were normalized to GAPDH and significant differences (*P* < 0.05) between sexes in co-ex are noted by an asterisk (^*^). **(B)** Similar analysis of the above genes was performed in KO males and females. Significant upregulation was observed for CCND1, CCND2, CCNE1, CDK7, IL-6, TLR2, TLR3, STAT3, MYD88, PTEN, TNFSF12, MTDH, JUN, E2F3, TNF, CDK2, SMAD2, and AKT1 in KO males, whereas the expression levels of IL-10, MMP2, ARG1, and PPARA were significantly upregulated in KO females. The expression levels were normalized to GAPDH and the significant differences (*P* < 0.05) between sexes in KO are noted by an asterisk (^*^).

To measure the expression levels of target genes by differentially expressed miRNA in response to FA or O_3_, we performed qRT-PCR analysis on AM cell samples isolated from co-ex and KO males and females after FA or O_3_ exposure (Figure 5).

In response to OxS, the expression level of all target genes studied, with the exception of SMAD2 that remained similar in both sexes under OxS, was significantly upregulated in co-ex males compared to females (Figure 5A, Supplementary Table 2). These included CCND1, CCND2, CCNE1, CDK7, IL-6, IL-10, TLR2, TLR3, STAT3, MYD88, IL-4, IL2RG, EGR2, PTEN, TNFSF12, MTDH, JUN, E2F3, BCL2, TNF, CDK2, MMP2, ARG1, AKT1, PPARA, and MYC. A similar analysis in KO males and females resulted in significant upregulation of CCND1, CCND2, CCNE1, CDK7, IL-6, TLR2, TLR3, STAT3, MYD88, PTEN, TN*F*SF12, MTDH, JUN, E2F3, TNF, CDK2, SMAD2, and AKT1 in KO males (Figure 5B, Supplementary Table 2), whereas the expression level of IL-10, MMP2, ARG1, and PPARA was significantly upregulated in KO females (Figure 5B). The levels of IL-4, IL2RG, EGR2, BCL2, and MYC remained similar in both sexes under OxS (Figure 5B).

In Summary the collective information of the significantly changed miRNAs and their targets indicate sex specific differences. Females (unlike males) largely failed to upregulate the target genes as expected when regulatory miRNAs are downregulated.

## Discussion

Surfactant protein-A (SP-A), is a key molecule in the lung innate immunity and surfactant related functions. The human SP-A locus consists of two functional genes, *SFTPA1* and *SFTPA2* (11, 12) and encodes two functional proteins, SP-A1 and SP-A2, respectively, and each is identified with several genetics variants (13, 14). Recently, it has been shown that SP-A1 and SP-A2 variants differ in their ability to regulate the AM miRNome in response to ozone (O_3_)-induced oxidative stress (OxS) (41) as well as in lung function mechanics and survival in response to bacterial infection (42, 43). Because humans express both SP-A gene products, we wished to investigate the combined effect of co-expressed SP-A1/SP-A2 (co-ex) in response to O_3_ induced OxS on AM miRNome. Toward this, human transgenic (hTG) mice, carrying both SP-A1/SP-A2 (6A^2^/1A^0^, co-ex) and SP-A-KO were exposed to filtered air (FA) and O_3_ and miRNA levels were measured after AM isolation with or without normalization to KO. The observation made include, (i) Significant differences in AM miRNome of co-ex in terms of sex, exposure, with or without normalization to KO, and after Bonferroni multiple comparison analysis; (ii) After normalization with KO, both males and females showed significant differences in response to OxS; (iii) The AM miRNome of females was largely down regulated significantly in response to OxS compared to control (FA) in all comparisons made including the multiple comparison analysis; (iv) The miRNA targets were largely downregulated in females and upregulated in males; (v) Several of the mRNA targets identified of the significantly altered miRNAs in females were involved in pro-inflammatory response, anti-apoptosis, cell cycle, cellular growth and proliferation; (vi) The AM of the SP-A2 male (41) shares similarities with the co-ex, as well as differences.

We studied the AM miRNome in co-ex male and female mice that express human SP-A1/SP-A2 (6A^2^/1A^0^, co-ex) after O_3_ exposure and compared it to that of KO mice lacking SP-A. In response to OxS, AM miRNome changes were observed in both males and females (with or without normalization to KO), although after multiple comparison analysis, AM miRNAs significantly changed only in females. Furthermore, IPA of the differentially expressed co-ex AM miRNome data identified several miRNA targets involved in the pro-inflammatory response, anti-apoptosis, cell cycle, and cellular growth and proliferation, as shown in Figure 4 and discussed below.

### Pro-inflammatory Responses

The expression of miR-191-5p, miR-155-5p and miR-92a-3p were decreased significantly in response to O_3_ in both co-ex males and females (Figures 4A,B). These miRNAs target pro- and anti-inflammatory IL-6 cytokine (41, 65–67). The IL-6 level decreased significantly in co-ex females, whereas this increased significantly in co-ex males (Figures 4A,B) compared to the control mRNA (GAPDH). The latter is consistent with our previous observation with SP-A2 males in response to OxS (41).

IL-6 plays a crucial role in the activation of STAT3 (68–70). In response to OxS, STAT3 gets phosphorylated which results in the activation of genes involved in inflammation and injury (71). miR-17-5p, and miR-1195 that were significantly altered in response to OxS, are known to interact with STAT3 (Figures 4A,B). In co-ex females and males miR-17-5p is downregulated whereas miR-1195 is downregulated in co-ex females but upregulated in co-ex males. Although, the downregulation of these miRNAs in females should have resulted in an increase in the expression of STAT3, the opposite was observed, indicating that either the regulation of STAT3 by these (and perhaps other) miRNAs is dysfunctional or mechanisms other than miRNAs are involved. The downregulation of STAT3 in females was associated with decreased levels of TNF, TNFSF12, IL-6, IL-10, and IL-4 (Figure 4B). In contrast, in male co-ex, although miR-17-5p is decreased, and miR-1195 is increased, STAT3, which is target for both, is increased, as well as the levels of the pro-inflammatory cytokines were increased as one may expect. Also in males the levels of a number of target genes (ARG1, EGR2, and IL2RG) that contribute, via STAT3, to the upregulation of pro-inflammatory cytokines were upregulated. Although, the details of the underlying mechanisms are unknown currently, these data show a disconnect between miRNA expression and target gene expression in females. The emerging picture is that pro-inflammatory cytokines are downregulated in females and upregulated in males (Figure 4A).

Toll like receptors (TLRs) are a family of membrane bound proteins that recognize pathogen-associated molecular patterns and mediate innate immune response (72). SP-A differentially regulates TLR expression (73). We found four co-ex regulated miRNAs (miR-92a-3p, miR-125b-5p, miR-155-5p, and miR-191-5p) that target TLR2, and TLR3. The expression of these miRNAs is significantly downregulated in our study in both males and females, and this is associated with increased mRNA levels of TLR2 and TLR3 in co-ex males but a decrease in co-ex females. Both miR-125b-5p and miR-155-5p are shown in several studies to regulate TLRs (74–77). TLR2 engages the ubiquitous intracellular adaptor MyD88 (myeloid differentiation primary response 88) and TLR3 engages TRIF (TIR-domain containing adaptor protein). In the current study, the level of miR-125b-5p, miR155-5p and miR130b-5 targeting MyD88 are downregulated and the mRNA level of MyD88 is upregulated in co-ex males but decreased in co-ex females. The involvement of TLR2, TLR3, and activation of MyD88 in co-ex males may result in the recruitment of other genes involved in the activation of NF-kB (78, 79), and this may result in the transcription of pro-inflammatory genes, such as TNF, TNFSF12, IL-10, and IL-4, as observed in the present study (Figures 4A,B). Previous studies have provided evidence that SP-A activates NF-kB (80) either through accumulation of inhibitory IkBa (81, 82) or via direct interaction with TLR2 and TLR4 (73, 80, 83–85) or SIRPα and CD91 (86). It has also been shown that, SP-A is unable to activate NF-kB in response to O_3_ as assessed by the lack of changes in the nuclear p65 subunit and the cytoplasmic IkBa levels as it would have been expected in the classical NF-kB pathway (87). Moreover, it has been shown that decreased levels of MTDH expression attenuate NF-kB signaling (88) and that TNFSF12 regulates NF-kB activity (89). Upregulation of TNFSF12 and MTDH as it occurs in co-ex males in the current study may alter NF-kB signaling, enhance its translocation to the nucleus to facilitate the transcription of pro-inflammatory genes in co-ex males but not in females. The present data support the idea that NF-kB and STAT3-mediated pathways play a role in the pro-inflammatory gene expression in co-ex males, and that these pathways in females are compromised in response to OxS.

### Anti-apoptosis, Cell Cycle, Growth, and Proliferation

The expression of two miRNAs miR-16-5p and miR-21a-5p (TargetScan) that bind BCL2 (90–93) was significantly downregulated in both males and females in response to OxS and the BCL2 mRNA levels were increased in co-ex males but decreased in females. An increase in BCL2 is likely to result in the inhibition of apoptosis and cell proliferation in males, but not in co-ex females (Figures 4A,B). These findings together indicate that OxS differentially affects anti-apoptotic pathways in co-ex males and females, and that females seem to have a disconnect between miRNA expression and target gene expression.

A number of miRNAs whose expression was for the most part downregulated significantly in response to OxS were predicted to target CCND1, CCND2, CCNE1, CDK2, CDK7, and E2F3 (Figures 4A,B). miR-16-5p and miR-17-5p, predicted to bind CCND1, CCND2, CCNE1, and E2F3 mRNAs (TargetScan), have been shown in several studies that these genes are regulated by these two miRNAs (94–97). The expression of all the target genes followed a similar pattern as described above, there was an increase in males and decrease in females. These genes are involved in cell cycle, and growth and proliferation, indicating that ozone differentially affects expression of molecules involved in cell cycle and proliferation pathways in co-ex male and female mice. Of interest significant differences in survival have been observed with females being more affected than males in several lung diseases (53–56). In our animal studies, we observed a significant difference in survival after infection and O_3_ exposure. Females were more susceptible to oxidative stress than males and exhibited lower survival (7, 9). Sex hormones were shown to play a role in the observed survival differences (57).

AKT, PPARA, PTEN, and JUN involved in the MAPK pathway were significantly upregulated in co-ex males but not in females in response to OxS. The miRNAs that targeted these genes, miR-21-5p, miR-103-3p, let-7a-5p, miR-130b-3p, miR-151-5p, and miR-92-3p, were significantly downregulated in co-ex males and females (Figures 4A,B). The upregulation of MAPK pathway genes has the potential to regulate the genes involved in cell cycle, growth and proliferation (as shown in Figure 4), as well as pro-inflammatory and anti-apoptotic genes (not shown) in co-ex males but not in co-ex females in response to OxS.

**Figure 6:**
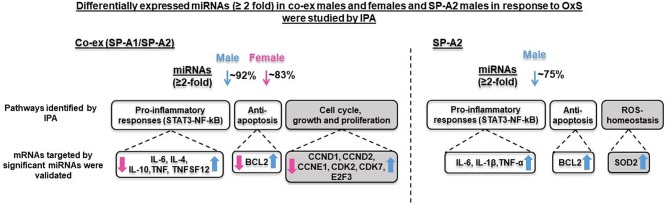
Overview of the effect of both gene (SP-A1/SP-A2) product and of single gene product (SP-A2) on the regulation AM miRNome on both males and females, and males respectively. The diagram depicts comparison of miRNAs, target genes, and pathways after OxS in AM from mice carrying both (co-ex) SP-A1/SP-A2 variants (male and female, left, current study) and from mice carrying a single SP-A2 (male) gene variant (right) (41). The SP-A2 work has been published (41) and is used here for comparison reasons only. The significantly regulated miRNAs (23 and 11) in co-ex male and female and in SP-A2 male, respectively, in response to OxS were largely decreased (~92% in co-ex male, ~83% in co-ex female, and ~75% in SP-A2 male) with the exception of few miRNAs that were increased (see Figure 4). All the mRNA targets were upregulated in co-ex male and in SP-A2 and are shown in blue arrow (on the right). The mRNA targets in co-ex females were downregulated and are shown in pink arrow (on the left). The pathways that differ in SP-A2 and co-ex in response to OxS are shown in gray background and those that are similar between co-ex males and SP-A2 male are shown in white background.

We have previously studied the effect of a single, SP-A1 or SP-A2 gene, on the AM miRNome after OxS, and found that SP-A2 (but not SP-A1) had a significant impact on AM in males (41). To gain further insight into the contributions of SP-A2 vs. the co-ex on AM miRNome, we compared the data from two studies. This comparison is shown diagrammatical in Figure 6. A number of observations are readily evident. Broadly these show (1) the miRNAs that changed (≥2 fold) significantly in response to OxS were decreased in male SP-A2 and co-ex male and female after normalization to KO. (2) In males (SP-A2 & co-ex) the expression of all validated target genes (except SMAD2), of the significant miRNAs, identified by IPA, is increased but decreased in co-ex females. (3) The common pathways include pro-inflammatory response, and anti-apoptosis. However the SP-A2 males include ROS-homeostasis processes and the co-ex include cell cycle, growth, and proliferation processes. These data show that SP-A2 alone or in combination with SP-A1 may regulate the expression of pro-inflammatory genes via the STAT3-NF-kB pathway and anti-apoptotic genes in response to OxS (Figure 4A). Furthermore, this indicated that these pathways may in part (if not in their entirety) be regulated or driven by SP-A2 and that the presence of SP-A1 does not negatively affect this. However, although SP-A1 by itself did not have any significant effect on AM, in presence of SP-A2 is shown to regulate genes involved in cell cycle, growth and proliferation, whereas SP-A2 alone regulates genes involved in homeostasis of ROS (41).

Although the data of this study are largely in line with our previous observations, the current study has a few limitations: (a) the study is carried out at single time point, (b) validation analysis was performed only for genes which are targeted by significantly changed miRNAs in co-ex males and females after normalizing to KO, and we did not look at the protein levels of the targeted mRNAs, (c) we did not study the molecular mechanisms of the identified pathways, (d) we did not differentiate the impact of varying amount SP-A1 and SP-A2 on miRNA expression in response to OxS. It has been shown that the ratio of SP-A1 to SP-A differs in lung diseases (23, 24), and this may have functional consequences given the varying activities of SP-A1 and SP-A2, (e) we did not study the impact of gonadectomy on the expression of AM miRNAs from co-ex and KO males and females. However, we have previously studied the effect of a single, SP-A1, or SP-A2 gene, on the AM miRNome after OxS, and found that SP-A2 (but not SP-A1) had a significant impact on AM in males (41). In this study, we observed that the regulation of the miRNome of the SP-A2 male mice compared to that of female mice in response to OxS is significantly altered after gonadectomy (41). It has also been shown that different stages of the estrous cycle have significant impact on the lung miRNA expression (98). In addition, a role of sex hormones on survival after *Klebsiella pneumoniae* infected wildtype (SP-A) mice with or without exposure to ozone has been observed (57). This study indicated that (1) after removal of gonadal hormones, differences in survival in animals after infection, and oxidative stress are minimized in males, and eliminated in females. (2) Treatment of gonadectomized females with DHT and males with E2 resulted in a similar kind of survival compared to the intact male and female animals, respectively. This further supports a role of DHT and E2 in survival after infection and oxidative stress. Based on these observations, we speculate that in co-ex males and females sex hormones play a significant role in the regulation of AM miRNome. However, the result of this study advances our knowledge of the differential impact of co-expressed SP-A1/SP-A2 and sex on the AM miRNome.

We postulate that in males in response to OxS, SP-A2 via its activity in ROS-homeostasis provides some protection from the injurious ROS in its microenvironment. Whereas, the co-ex males via cell cycle, growth, and proliferation process may promote cellular recovery, perhaps a more sustained recovery. We further postulate that in co-ex females the disconnect between the downregulation of miRNAs and the expression of their target genes is responsible or contributes to the reduced ability in females to enhance phagocytosis by AM as well as to the poorer survival we observed in females after OxS and infection (9). Although the details of the underlying mechanisms are unknown, the AM miRNome appears to play a significant role in OxS.

In summary, SP-A1/SP-A2 (6A^2^/1A^0^, co-ex) regulate miRNAs that play a role in pathways involved in inflammatory responses, anti-apoptosis, cell cycle, growth, and proliferation. Both gene products are needed to alleviate the deleterious effects of OxS in males and promote cellular recovery. However, in females even in the presence of both SP-A1 and SP-A2 genes, expression of target genes to mitigate the OxS injury is lacking, indicating that other hormone dependent mechanisms are involved. Because the innate immune molecules, SP-A1 and SP-A2 appear to play a differential role in the outcome of males and females after OxS, the potential impact on health of innate immune genetics should be considered separately in males and females.

## Data Availability

The datasets generated for this study are included in the manuscript and the Supplementary Files, and has been deposited in the Gene Expression Omnibus repository GSE135233 (https://www.ncbi.nlm.nih.gov/geo/query/acc.cgi?&acc=GSE135233).

## Ethics Statement

All protocols used in this study was evaluated and approved by the Pennsylvania State University College of Medicine Institutional Animal Care and Use Committee and Confirmed to the guidelines of the National Institute of Health on the care and use of laboratory animals.

## Author Contributions

NT performed experiments, run statistics, analyzed and synthesized the data, contributed to the manuscript writing. YK performed RNA sequencing analysis. CG contributed to the manuscript writing. XZ performed mouse line maintenance, breeding. JF designed the study and provided oversight to the entire project, involved in data analysis, integration, and writing of the manuscript. All authors read and approved the final manuscript.

### Conflict of Interest Statement

The authors declare that the research was conducted in the absence of any commercial or financial relationships that could be construed as a potential conflict of interest.

## Supplementary Material

Supplementary Table 1 Identification of miRNAs from SP-A1/SP-A2 (co-ex), KO males and females in response to Filtered air or Ozone.

Supplementary Table 2 Expression levels of target genes by differentially expressed miRNAs in response to FA or Ozone by qRT-PCR.

## Abbreviations

AKT1: AKT Serine/Threonine Kinase 1
ARG1: Arginase 1
AM: Alveolar macrophages
ANOVA: analysis of variance
BAL: bronchoalveolar lavage
BCL2: B-cell lymphoma 2
CCND1: Cyclin D1
CCND2: Cyclin D2
CCNE1: Cyclin E1
CDK2: Cyclin-dependent kinase 2
CDK7: Cyclin-dependent kinase 7
E2F3: E2F transcription factor 3
EGR2: Early growth response 2
FA: Filtered air
GAPDH: Glyceraldehyde 3-phosphate dehydrogenase
hTG: Humanized transgenic
IkBa: NFKB Inhibitor Alpha
IL4: Interleukin 4
IL6: Interleukin 6
IL10: Interleukin 10
IL2RG: Interleukin 2 receptor subunit gamma
IPA: Ingenuity Pathway Analysis
JUN: Jun proto-oncogene
KO: knock-out
LPS: lipopolysaccharide
MAPK: Mitogen-activated protein kinases
MDTH: Metadherin
miRNAs: microRNAs
MMP2: Matrix metallopeptidase 2
MYC: MYC proto-oncogene
MYD88: Myeloid differentiation primary response 88
NF-kB: Nuclear factor kappa-light-chain-enhancer of activated B cells
O_3_: ozone
OxS: oxidative stress
PPARA: Peroxisome proliferator activated receptor alpha
PTEN: Phosphatase and tensin homolog
ROS: reactive oxygen species
S*FTPA*1: gene encoding SP-A1
S*FTPA2*: gene encoding SP-A2
SMAD2: SMAD family member 2
SP-A: surfactant protein A
STAT3: Signal transducer and activator of transcription 2
TLR2: Toll-like receptor 2
TLR3: Toll-like receptor 3
TNF: Tumor necrosis factor
TNFSF12: TNF super family member 12
TRIF: TIR-domain containing adaptor protein.
